# Autoimmune Pancreatitis in Patients with Inflammatory Bowel Disease: A Real-World Multicentre Collaborative ECCO CONFER Study

**DOI:** 10.1093/ecco-jcc/jjad097

**Published:** 2023-06-07

**Authors:** Piotr Eder, Bram Verstockt, Emma Culver, Gabriele Dragoni, Lea Isabell Kredel, Joanna Wypych, Ana Garcia Garcia de Paredes, Magdalena Kaniewska, Haim Leibovitzh, Triana Lobaton, Marie Truyens, Grzegorz Oracz, Davide Giuseppe Ribaldone, Teresa Starzyńska, Abdenor Badaoui, Jean-Francois Rahier, Cristina Bezzio, Peter Bossuyt, Katherine Falloon, Daniela Pugliese, Catherine Frakes Vozzo, Tine Jess, Lone Larsen, Søren Schou Olensen, Partha Pal, María Chaparro, Dikla Dror, Pierre Ellul, Iga Gromny, Maria Janiak, Katarzyna Maciejewska, Noam Peleg, Ariella Bar-Gil Shitrit, Łukasz Szwed, Renata Talar-Wojnarowska, Yifat Snir, Roni Weisshof, Eran Zittan, Izabela Miechowicz, Idan Goren

**Affiliations:** Department of Gastroenterology, Dietetics, and Internal Medicine, Poznan University of Medical Sciences, Poznan, Poland; Department of Gastroenterology and Hepatology, University Hospitals Leuven, KU Leuven, Leuven, Belgium; Department of Chronic Diseases and Metabolism, KU Leuven, Leuven, Belgium; Translational Gastroenterology Unit, John Radcliffe Hospital and Oxford, NIHR BRC, Nuffield Department of Medicine, University of Oxford, Oxford, UK; Department of Gastroenterology, Careggi University Hospital, Florence, Italy; Division of Gastroenterology, Infectiology and Rheumatology, Medical Department, Charité-Universitätsmedizin, Berlin, Germany; Department of Gastroenterology, Surgery and Nutrition, Copernicus Hospital, Gdansk, Poland; Gastroenterology and Hepatology Department. Hospital Universitario Ramon y Cajal. Universidad de Alcala, IRYCIS, Madrid, Spain; Department of Gastroenterology with IBD Subdivision, National Medical Institute of Ministry of Inferior and Administration, Warsaw, Poland; Zane Cohen Centre for Digestive Diseases, Division of Gastroenterology & Hepatology, Temerty Faculty of Medicine, Mount Sinai Hospital, University of Toronto, Toronto, Ontario, Canada; Department of Internal Medicine and Pediatrics, Department of Gastroenterology, Ghent University, Ghent, Belgium; Department of Internal Medicine and Pediatrics, Department of Gastroenterology, Ghent University, Ghent, Belgium; Department of Gastroenterology, Hepatology, Feeding Disorder and Pediatrics, The Children’s Memorial Health Institute, Warsaw, Poland; Pediatric Gastroenterology Faculty, Centre of Postgraduate Medical Education, Warsaw, Poland; Department of Medical Sciences, University of Turin, Turin, Italy; Department of Gastroenterology, Pomeranian Medical University in Szczecin, Szczecin, Poland; Department of Gastroenterology, Université Catholique de Louvain, Yvoir, Belgium; Department of Gastroenterology, Université Catholique de Louvain, Yvoir, Belgium; Gastroenterology Unit, Rho Hospital, Rho (MI), ASST Rhodense, Garbagnate Milanese, Italy; Imelda GI Clinical Research Center, Imelda General Hospital, Bonheiden, Belgium; Department of Gastroenterology, Hepatology and Nutrition, Digestive Diseases and Surgery Institute, Cleveland Clinic, Cleveland, OH, USA; CEMAD, IBD UNIT, Unità Operativa Complessa di Medicina Interna e Gastroenterologia, Dipartimento di Scienze Mediche e Chirurgiche, Fondazione Policlinico Universitario ‘A. Gemelli’ IRCCS, Rome, Italy; Department of Gastroenterology, Hepatology and Nutrition, Digestive Diseases and Surgery Institute, Cleveland Clinic, Cleveland, OH, USA; Center for Molecular Prediction of Inflammatory Bowel Disease, Department of Clinical Medicine, Aalborg University, Copenhagen, Denmark; Department of Gastroenterology and Hepatology, Aalborg University Hospital, Center for Molecular Prediction of Inflammatory Bowel Disease – PREDICT, Department of Clinical Medicine, The Faculty of Medicine, Aalborg University, Aalborg, Denmark; Centre for Pancreatic Diseases and Mech-Sense, Department of Gastroenterology and Hepatology, Aalborg University Hospital, Aalborg, Denmark; Department of Clinical Medicine, Aalborg University, Aalborg, Denmark; Department of Gastroenterology, Asian Institute of Gastroenterology, Hyderabad, India; Department of Gastroenterology, Hospital Universitario de La Princesa, Instituto de Investigación Sanitaria Princesa (IIS-Princesa), Universidad Autónoma de Madrid (UAM), and Centro de Investigación Biomédica en Red de Enfermedades Hepáticas y Digestivas (CIBEREHD), Madrid, Spain; Department of Gastroenterology, Galilee Medical Center, Nahariyya, Israel; Division of Gastroenterology, Mater dei Hospital, Malta; Division of Dietetics, Department of Gastroenterology and Hepatology, Wroclaw Medical University, Wroclaw, Poland; Department of Gastroenterology and Hepatology, Medical University of Gdańsk, Gdańsk, Poland; Department of Gastroenterology with IBD Subdivision, National Medical Institute of Ministry of Inferior and Administration, Warsaw, Poland; The Division of Gastroenterology, Rabin Medical Center, Petach Tikva, Israel, affiliated with Sackler Faculty of Medicine, Tel Aviv University, Tel Aviv, Israel; IBD MOM Unit, Digestive Diseases Institute, The Hebrew University of Jerusalem, Shaare Zedek Medical Center, Jerusalem, Israel; Private Gastroenterology Practice, Nowy Dwór Mazowiecki, Poland; Department of Digestive Tract Diseases, Medical University of Lodz, Lodz, Poland; Gastroenterology Department, Clalit Health Services, Tel Aviv District, affiliated with Sackler Faculty of Medicine, Tel Aviv University, Tel Aviv, Israel; Gastroenterology Institute at Rambam Health Care Campus in Haifa, Haifa, Israel; Ellen and Pinchas Mamber Institute of Gastroenterology and Liver Diseases, IBD Unit, Emek Medical Center, Afula, Israel; Department of Computer Science and Statistics, Poznan University of Medical Sciences, Poznan, Poland; Department of Inflammation and Immunity, Lerner Research Institute, Cleveland Clinic, Cleveland, OH, USA

**Keywords:** Autoimmune pancreatitis, inflammatory bowel disease, pancreatic insufficiency

## Abstract

**Background:**

Autoimmune pancreatitis [AIP] is rarely associated with inflammatory bowel disease [IBD]. The long-term outcomes of AIP and IBD in patients with coexisting AIP–IBD and predictors of complicated AIP course have rarely been reported.

**Methods:**

An ECCO COllaborative Network For Exceptionally Rare case reports project [ECCO-CONFER] collected cases of AIP diagnosed in patients with IBD. Complicated AIP was defined as a composite of endocrine and/or exocrine pancreatic insufficiency, and/or pancreatic cancer. We explored factors associated with complicated AIP in IBD.

**Results:**

We included 96 patients [53% males, 79% ulcerative colitis, 72% type 2 AIP, age at AIP diagnosis 35 ± 16 years]. The majority of Crohn’s disease [CD] cases [78%] had colonic/ileocolonic involvement. In 59%, IBD preceded AIP diagnosis, whereas 18% were diagnosed simultaneously. Advanced therapy to control IBD was used in 61% and 17% underwent IBD-related surgery. In total, 82% of patients were treated with steroids for AIP, the majority of whom [91%] responded to a single course of treatment. During a mean follow-up of 7 years, AIP complications occurred in 25/96 [26%] individuals. In a multivariate model, older age at AIP diagnosis was associated with a complicated AIP course (odds ratio [OR] = 1.05, *p* = 0.008), whereas family history of IBD [OR = 0.1, *p* = 0.03], and CD diagnosis [OR = 0.2, *p* = 0.04] decreased the risk of AIP complications. No IBD- or AIP-related deaths occurred.

**Conclusions:**

In this large international cohort of patients with concomitant AIP–IBD, most patients have type 2 AIP and colonic IBD. AIP course is relatively benign and long-term outcomes are favourable, but one-quarter develop pancreatic complications. Age, familial history of IBD, and CD may predict uncomplicated AIP course.

## Graphical Abstract

**Graphical Abstract:**
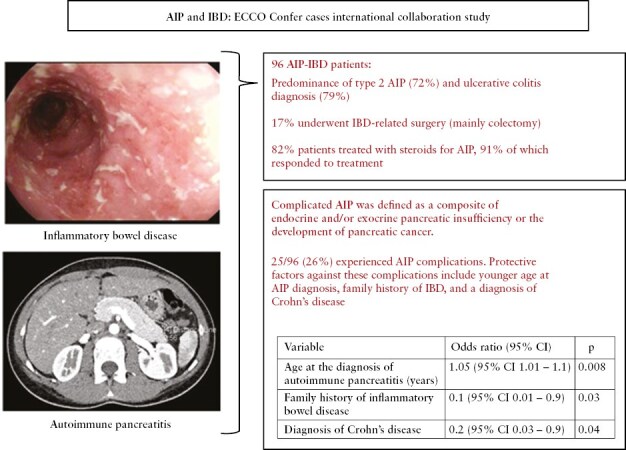

## 1. Introduction

Inflammatory bowel disease [IBD] is a systemic disorder frequently associated with extraintestinal manifestations and concomitant immune-mediated diseases.^1^ Autoimmune pancreatitis [AIP] is a rare inflammatory disorder of unknown origin.^2–4^ AIP is classically divided into two subtypes: type 1 AIP, which is the pancreatic manifestation of a systemic inflammatory IgG4-related disorder,^3^ and type 2 AIP, which is a selective fibroinflammatory pancreatic disease not related to IgG4.^2^

AIP has previously been reported in association with IBD.^4–9^ However, its exact prevalence might be underestimated due to the lack of systemic manifestations or specific serum markers such as IgG4 in the majority of cases. Specifically, type 2 AIP mainly affects young patients and one-third of those with concomitant IBD.^10^ As a result, symptoms arising from pancreatic inflammation, such as abdominal pain, can be misinterpreted as a manifestation of the IBD rather than a pancreatic disorder. Moreover, steroids, being in many cases the first therapeutic choice in IBD flare, are also effective induction therapy in type 2 AIP with a low relapse rate.^4,5^

Little is known about the clinical course, long-term response to treatment, and the prognosis of both conditions in patients with concomitant IBD and AIP. In this study, we aimed to describe an international series of patients with IBD and AIP and to identify risk factors for AIP complications.

## 2. Patients and Methods

### 2.1. Study design

This was an observational multicentre retrospective study initiated through the European Crohn’s and Colitis Organisation [ECCO] COllaborative Network For Exceptionally Rare [CONFER] cases. The CONFER project was initiated by ECCO to specifically identify and report together rare IBD disease associations, which otherwise are seldom reported due to their exceptional rarity.^11^ Once a specific topic was selected by the steering committee, ECCO launched a call to identify similar cases encountered by IBD physicians worldwide. The call to physicians was made through announcements at the ECCO annual congress and in national and international IBD meetings. Furthermore, the call for similar cases was disseminated by direct emails to all ECCO members and affiliated physicians and on the ECCO website and eNews. Physicians were then prompted to report their cases to the CONFER database using a standardized case reporting form.

### 2.2. Patients and procedures

Adult patients diagnosed with IBD and concomitant AIP were eligible for inclusion in this project. Diagnosis of AIP was based on the combination of clinical, biochemical [such as level of serum IgG4 and autoantibodies], radiological, and/or histological criteria after exclusion of other aetiologies of pancreatic disorders.^3^ The case report form was divided into two sections. Section 1 included patient [epidemiological data, past medical history, smoking, family history] and IBD characteristics [date of diagnosis, Montreal classification, extraintestinal manifestations, treatments, and surgery for IBD]. Section 2 included a description of AIP characteristics [date of diagnosis, presenting symptoms, AIP type, diagnostic criteria and diagnostic modalities, therapy and response to therapy, AIP-related endocrine and exocrine complications, and mortality at the time of the last follow-up visit]. The definition of response to treatment in our study was based on long-term improvement of clinical symptoms reported by the patients and confirmed by the treating physician. Complicated AIP was defined as a combination of endocrine and/or exocrine pancreatic insufficiency or the occurrence of pancreatic cancer. Data were collected and analysed anonymously and handled according to local regulations.

### 2.3. Statistical analysis

Categorical variables are described as frequency and percentage. The distribution of normality of the variables was tested with the Shapiro-Wilk test. To analyse the variables between patients with complicated AIP compared to those with an uncomplicated AIP course, we used the Mann–Whitney test. The relationship between categorical variables was calculated using the chi-square test, the Fisher exact test, or the Fisher–Freeman–Halton test. We then constructed a logistic regression model to investigate predictors for complicated AIP. The statistical significance of individual model variables was tested with the Wald chi-square test and the significance of the model with the likelihood ratio test. The sensitivity and specificity, as well as the negative and positive likelihood ratios with 95% confidence intervals [CI] of the model, were calculated and the receiver operating curve [ROC] was determined. The calculations were made using Statistica v.13 by TIBCO and PQStat v.1.8.4.136 by PQStat software. The level of significance was α = 0.05. The result was considered statistically significant when *p* < α.

### 2.4. Ethical considerations

The study was approved by the local institutional review boards of the participating centres if required, according to local regulations. Due to the retrospective and anonymized nature of the data, the requirement for informed consent was waived. The investigators and the participating sites treated all information and data related to the study as confidential and the disclosed information was not used for any purpose other than the performance of the study.

## 3. Results

### 3.1. Study population

Ninety-six patients from 37 centres in 12 countries [Supplementary Table S1] were enrolled in the study. Of the 96 patients included, 51 [53%] were males [Table 1]. The mean (± standard deviation [SD]) age at IBD diagnosis was 32 ± 15 years. Seventy-six patients [79%] were diagnosed with ulcerative colitis [UC], 18 [19%] with Crohn’s disease [CD], and two [2%] with IBD unclassified [IBDU]. Of the 18 patients with CD, 14 [78%] had colonic or ileocolonic involvement. In 57 [59%] patients, IBD diagnosis preceded that of AIP, whereas in 22 [23%] cases AIP preceded IBD diagnosis, and in 17 [18%] both conditions were diagnosed simultaneously.

**Table 1.** Characteristics of the study group of patients with coexisting autoimmune pancreatitis and inflammatory bowel disease.

Feature	Entire cohort [*N* = 96]
Gender [males]	51 [53%]
Current age [years ± SD]	40 ± 16
Age at IBD diagnosis [years ± SD]	32 ± 15
Duration of IBD follow-up [years ± SD]	7 ± 6
Type of IBD, *n* [%]	
Crohn’s disease	18 [19%]
Ulcerative colitis	76 [79%]
IBDU	2 [2%]
Montreal IBD classification, *n* [%]	
Age at CD diagnosis ≤ 16 years	3/18 [17%]
Age at CD diagnosis 17–40 years	7/18 [39%]
Age at CD diagnosis > 40 years	8/18 [44%]
Ileal CD [L1]	2/18 [11%]
Colonic CD [L2]	7/18 [39%]
Ileocolonic CD [L3]	7/18 [39%]
Upper gastrointestinal CD involvement [L4]	2/18 [11%]
CD inflammatory phenotype [B1]	16/18 [89%]
CD stricturing phenotype [B2]	2/18 [11%]
CD penetrating phenotype [B3]	0/18 [0%]
Perianal CD	2/18 [11%]
UC E1	10/76 [13%]
UC E2	35/76 [46%]
UC E3	31/76 [41%]
EIM, *n* [%]	21 [22%]
IBD-related interventions [past and present], *n* [%]	
Systemic steroids	74 [77%]
Mesalamine	75 [78%]
Immunomodulators	48 [50%]
Biologics^a^	41 [43%]
IBD-related surgery^b^	16 [17%]
Number of IBD flares, median [IQR]	2 [1–4]
Comorbidities, *n* [%]	39 [41%]
Family history of IBD, *n* [%]	19 [20%]
Active IBD status at the end of follow-up, *n* [%]	14 [15%]
Age at AIP diagnosis [years ± SD]	35 ± 16
Duration of AIP follow-up [years ± SD]	5 ± 4
Predominant symptoms at the onset of AIP, *n* [%]	
Abdominal pain	78 [81%]
Jaundice	4 [4%]
Weight loss	3 [3%]
Asymptomatic	10 [11%]
AIP subtype, *n* [%]	
1	19 [20%]
2	69 [72%]
Undefined	8 [8%]
AIP radiological subtype, *n* [%]	
Focal AIP presentation in imaging	39 [41%]
Diffuse AIP presentation in imaging	45 [47%]
Timing of diagnosis	
AIP diagnosis preceded IBD, *n* (%)	22 [23%]
IBD diagnosis preceded AIP or concomitant diagnosis, *n* [%]	74 [77%]
AIP-related treatments and course, *n* [%]	
Single steroid course with clinical response	72/79 [91%]
Steroid refractory	7/79 [9%]
Advanced therapy^c^	18 [19%]
Number of AIP relapses, mean ± SD	0.4 ± 0.9
Active AIP status at the end of follow-up, *n* [%]	5 [5%]

^a^Infliximab, adalimumab, golimumab, certolizumab, vedolizumab, ustekinumab, etrolizumab.

^b^Colectomy [*n* = 15], segmental intestinal resection [*n* = 1].

^c^Immunomodulator or biological therapy

Abbreviations: AIP—autoimmune pancreatitis, CD—Crohn’s disease, EIM—extraintestinal manifestations, IBD—inflammatory bowel disease, IBDU—inflammatory bowel disease unclassified, UC—ulcerative colitis.

The rate of active smokers was 10%. In total, 61% of patients [59 out of 96] required advanced therapy to control their underlying IBD [48/96 immunomodulators, 41/96 biologics] [Table 1]. Sixteen patients underwent IBD-related surgery, most frequently colectomy [15 in 76 UC patients; 20%].

### 3.2. AIP diagnosis and course

In about half of the cases [51 out of 96, 53%] AIP was diagnosed according to the combination of clinical, radiological, histological, and/or serological criteria without strict adherence to a specific formal diagnostic criterion. Of the remaining 45 patients [47%], the International Consensus Diagnostic Criteria [ICDC] were applied most frequently [*n* = 35]. The HISORt and the Asian criteria were used in eight and two centres, respectively.

Nineteen patients [20%] were considered to have AIP type 1, whereas 69 [72%] had AIP type 2. Eight patients [8%] were diagnosed with an undefined type of AIP.

Nine patients [9%] with type 1 AIP presented with extra-pancreatic manifestations, with cholangitis being the most common [7/9; 78%].

All patients underwent radiological assessment at the time of diagnosis, including magnetic resonance [63%], computed tomography [53%], or endoscopic ultrasound [45%]. Forty-five [47%] patients were diagnosed with diffuse type and 39 [41%] with focal AIP, whereas the remaining 12% had no defined radiological subtype.

As opposed to the frequent use of cross-sectional imaging techniques, only 40 patients [42%] underwent histological assessment of the pancreas. Of those patients, the most commonly used modality was endoscopic ultrasound-guided fine needle biopsy [EUS-FNB] (23/40 patients [58%]), followed by surgical resection [pancreaticoduodenectomy] due to suspected pancreatic cancer (7/40 patients [17%]) [Supplementary Table S2]. Of those undergoing histological assessment, a definitive histological diagnosis was obtained in 15/40 [38%]: 9/40 [23%] had lymphoplasmacytic sclerosing pancreatitis [LPSP] and 6/40 [15%] had idiopathic duct-centric pancreatitis [IDCP]. The remaining cases (25/40 [62%]) received a histological diagnosis suggestive of AIP without specifying the subtype.

Of 96 patients, 79 [82%] received steroids as the primary treatment, and 72 of those patients [91%] responded well to this therapy. The remaining 17 patients [18%] did not receive steroids initially. In most cases, no treatment was necessary due to the asymptomatic course of AIP or self-resolution of disease symptoms [*n* = 8]. Additionally, four patients in this subgroup underwent surgery due to suspicion of pancreatic cancer and did not require further treatment.

During long-term follow-up, 31 patients [32%] experienced at least one relapse of AIP and required one or more course of steroids or advanced treatment [immunodulator and/or biologics]. Eighteen out of 96 patients [19%] received immunosuppressive and/or biological treatment for controlling the AIP [thiopurines in 16 cases, methotrexate in one case, and/or anti-tumour necrosis factor alpha antibodies in three cases]. Notably, the use of advanced treatments aimed to control both AIP and active IBD in the majority of cases [16 out of 18]. Interestingly, none of the patients were treated with rituximab. Supplementary Table S3 presents the clinical efficacy of advanced treatment for AIP. At the end of the follow-up period, five patients [5%] were considered to have a clinically active AIP despite treatment.

### 3.3. AIP complications

During a mean [± SD] AIP follow-up of 5 ± 4 years, 25 patients [26%] developed complicated AIP, including exocrine pancreatic insufficiency [*n* = 19; 20%] and diabetes [*n* = 11; 11%]. One patient developed portal vein thrombosis and another patient developed common bile duct stenosis, both of whom had concomitant exocrine pancreatic insufficiency. There were no cases of pancreatic cancer.

**Figure 1.**
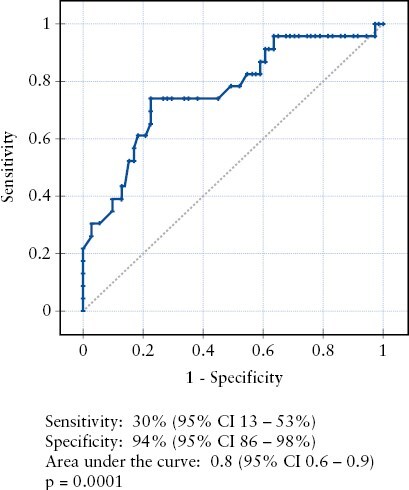
Receiver operator curve illustrating the ability of a proposed multivariate model to predict complicated autoimmune pancreatitis in a cohort of inflammatory bowel disease patients.

We then analysed baseline characteristics to explore possible associations with complicated AIP. Younger age at IBD or AIP diagnosis, no need for steroids to treat IBD, and family history of IBD were associated with an uncomplicated AIP course [Table 2]. The age cut-off of 32 years at AIP diagnosis best predicted the risk for developing AIP complications [*p* = 0.01, relative to ≥32 years], with a sensitivity, specificity, and area under the curve of 72%, 58% and 0.66 [95% CI 0.52–0.8], respectively. Finally, we performed a multivariate logistic regression model to control for potential confounders. We found that older age at AIP diagnosis increased the risk of developing complicated AIP (odds ratio [OR] 1.05; 95% CI 1.01–1.1; *p* = 0.008], whereas family history of IBD [OR 0.1; 95% CI 0.01–0.9; *p* = 0.03], and diagnosis of CD [OR 0.2; 95% CI 0.03–0.9; *p* = 0.04] were associated with a lower risk of developing pancreatic complications [Table 3]. This model was able to predict complicated AIP with a sensitivity of 30% [95% CI 13–53%] and specificity of 94% [95% CI 86–98%] with statistical significance [*p* = 0.0001] and an area under the curve of 0.8 [95% CI 0.6–0.9] [Figure 1]. The negative and positive likelihood ratios of the model were 0.7 [95% CI 0.6–0.9] and 5.4 [95% CI 1.7–16.8], respectively.

**Table 2.** Univariate analysis of risk factors associated with the development of complications in autoimmune pancreatitis among patients with inflammatory bowel disease.

Feature	AIP with complication, *n* = 25/96 [26%]	AIP without complications, *n* = 71/96 [74%]	*p*-value
Gender [males]	17 [68%]	34 [48%]	0.083
Current age [years ± SD]	49 ± 19	37 ± 14	**0.005**
Age at IBD diagnosis [years ± SD]	39 ± 19	30 ± 13	**0.031**
Duration of IBD follow-up [years ± SD]	9 ± 8	7 ± 6	0.090
Type of IBD, *n* [%]			
Crohn’s disease	2/18 [11%]	16/18 [89%]	**0.023** *
Ulcerative colitis	21/76 [28%]	55/76 [72%]	
IBDU	2/2 [100%]	0/2 [0%]	
Montreal IBD classification, *n* [%]			
Age at CD diagnosis ≤ 16 years	0/18 [0%]	3/18 [17%]	0.160
Age at CD diagnosis 17–40 years	0/18 [0%]	7/18 [39%]	
Age at CD diagnosis > 40 years	2/18 [11%]	6/18 [33%]	
Ileal CD [L1]	0/18 [0%]	2/18 [11%]	0.999
Colonic CD [L2]	2/18 [11%]	5/18 [28%]	
Ileocolonic CD [L3]	1/18 [5%]	6/18 [34%]	
Upper gastrointestinal CD involvement [L4]	0/18 [0%]	2/18 [11%]	
CD inflammatory phenotype [B1]	4/18 [22%]	12/18 [67%]	0.999
CD stricturing phenotype [B2]	0/18 [0%]	2/18 [11%]	
CD penetrating phenotype [B3]	0/18 [0%]	0/18 [0%]	
Perianal CD	0/18 [0%]	2/18 [11%]	0.999
UC E1	2/76 [3%]	8/76 [10%]	0.885
UC E2	11/76 [14%]	24/76 [32%]	
UC E3	8/76 [10%]	23/76 [31%]	
EIM, *n* [%]	7 [28%]	14 [20%]	0.389
IBD-related interventions [past and present], *n* [%]			
Systemic steroids	23 [92%]	51 [72%]	**0.039**
Mesalamine	20 [80%]	55 [77%]	0.792
Immunomodulators	12 [48%]	36 [51%]	0.816
Biologics^a^	11 [44%]	30 [42%]	0.879
IBD-related surgery^b^	7 [28%]	9 [13%]	0.116
Number of IBD flares, median [IQR]	3 [1–5]	2 [1–3]	0.058
Comorbidities, *n* [%]	14 [56%]	25 [35%]	0.069
Family history of IBD, *n* [%]	1 [4%]	18 [25%]	**0.021**
Active IBD status at the end of follow-up, *n* [%]	2 [8%]	12 [17%]	0.344

Age at AIP diagnosis [years ± SD]	42 ± 18	32 ± 14	**0.019**
Duration of AIP follow-up [years ± SD]	6 ± 5	5 ± 4	0.478
Predominant symptoms at the onset of AIP, *n* [%]			
Abdominal pain	18 [72%]	61 [86%]	0.232
Jaundice	2 [8%]	2 [3%]	0.277
Weight loss	2 [8%]	1 [1%]	0.165
Asymptomatic	3 [12%]	7 [10%]	0.717
AIP subtype, *n* [%]			
1	4 [16%]	15 [21%]	0.925
2	19 [76%]	50 [70%]	
Undefined	2 [8%]	6 [8%]	
AIP radiological subtype, *n* [%]			
Focal AIP presentation in imaging	11 [44%]	28 [39%]	0.690
Diffuse AIP presentation in imaging	11 [44%]	34 [48%]	0.738
Timing of diagnosis			
AIP diagnosis preceded IBD, *n* [%]	8 [32%]	14 [20%]	0.209
IBD diagnosis preceded AIP or concomitant diagnosis, *n* [%]	17 [68%]	57 [80%]	0.209
AIP-related treatments and course, *n* [%]			
Single steroid course with clinical response	15/17 [88%]	57/62 [92%]	0.639
Steroid refractory	2/17 [12%]	5/62 [8%]	0.639
Advanced therapy^c^	6 [24%]	12 [17%]	0.552
Number of AIP relapses, mean ± SD	0.5 ± 1	0.3 ± 0.9	0.368
Active AIP status at the end of follow-up, *n* [%]	1 [4%]	4 [6%]	<1.000

^a^Infliximab, adalimumab, golimumab, certolizumab, vedolizumab, ustekinumab, etrolizumab.

^b^Colectomy [*n* = 15], segmental intestinal resection [*n* = 1].

^c^Immunomodulator or biological therapy.

Abbreviations: AIP—autoimmune pancreatitis, CD—Crohn’s disease, EIM—extraintestinal manifestations, IBD—inflammatory bowel disease, IBDU—inflammatory bowel disease unclassified, UC—ulcerative colitis.

The differences were calculated by using chi-square, Fisher’s exact, or Fisher–Freeman–Halton tests for categorical variables. Continuous variables were compared by using the Mann–Whitney test due to non-compliance with the normal distribution.

*p values after Bonferroni correction: CD vs UC—*p* = 0.668; CD vs IBDU—*p* = 0.095; UC vs IBDU—*p* = 0.253.

**Table 3.** Multivariate logistic regression analysis of risk factors associated with the development of complications in autoimmune pancreatitis among patients with inflammatory bowel disease.

Variable	Odds ratio [95% confidence interval]	*p*-value
Age at diagnosis of autoimmune pancreatitis [years]	1.05 [1.01–1.1]	0.008
Family history of inflammatory bowel disease	0.1 [0.01–0.9]	0.03
Diagnosis of Crohn’s disease	0.2 [0.03–0.9]	0.04

## 4. Discussion

This international cohort showed that AIP type 2 is predominant in patients with IBD, with UC being the most frequent type of IBD. We demonstrated that IBD–AIP patients have a high risk of undergoing colectomy for active IBD. In contrast, the AIP course is relatively benign and the majority of patients respond initially to steroids. Nevertheless, in the long term, one-quarter of patients experienced endocrine and/or exocrine pancreatic insufficiency. We showed that younger age at AIP diagnosis, diagnosis of CD, and family history of IBD are protective factors that are independently associated with a lower risk of developing AIP complications among patients with IBD and AIP.

Our cohort demonstrates the heterogeneity in the diagnostic approaches for AIP in patients with IBD across different centres and countries. Only 40 patients underwent histological assessment and a total of 16% of the entire cohort met the histological criteria for definitive AIP diagnosis. This finding corroborates a previous report from the GETAID-AIP French study group, demonstrating that only 14 out of 91 [16%] participants had a definitive AIP diagnosis with histological confirmation.^9^ Moreover, our study shows that even when invasive modalities are utilized [i.e. EUS-FNB], a definitive histological diagnosis of LPSP or IDCP was rare. This may result from either the low quality of histological specimens^12^ or the shortage of specialized gastrointestinal pathologists, especially given the rarity of this entity. Other techniques, such as EUS-guided Trucut biopsy, are associated with a higher risk of technical failure and a slightly increased risk of complications.^12,13^ Therefore, our results show that when a benign condition, such as AIP, is suspected, a routine application of more invasive techniques is uncommon. Notably, in our series, 7% of patients underwent surgical resection for a suspected pancreatic malignancy at the time of diagnosis. Whether newer techniques, such as the ProCore® or SharkCore® needles, can improve the diagnostic yield of EUS-guided biopsy and defer surgery in such cases remain to be determined.^12,14,15^ Nevertheless, each case of an IBD patient with any pancreatic involvement should be carefully discussed by a multidisciplinary team including gastroenterologists, radiologists, pancreas specialists, and surgeons. Due to the lack of any surrogate diagnostic markers for AIP in the majority of IBD patients, histological assessment seems to be crucial, especially in the case of clinical, biochemical, and/or radiological worrisome features [Supplementary Table S4].^3,8,16,17^ Moreover, according to the consensus of the International Study Group of Pancreatic Surgery, when the probability of pancreatic cancer is high, the patient should be referred to surgery even if the histological assessment is not conclusive or, in selected cases, even without histological evaluation.^18^

Little is known about the characteristics of IBD in patients with concomitant AIP. Our results confirm that the majority of affected individuals have UC. This is in accordance with previous European cohorts from France,^9^ Sweden, and Italy.^19^ Interestingly, patients with CD and AIP represented a unique phenotype with a predominance of isolated colonic or ileocolonic locations [almost 80% of the CD subgroup] and with no perianal involvement. These findings are in line with those reported by Lorenzo *et al*. in a case-control study.^9^

Interestingly, some similarities between the clinical characteristics of AIP–IBD and primary sclerosing cholangitis [PSC]–IBD coexistence can be noted.^20^ They include a predominantly colonic IBD location, low rate of penetrating complications, and pancreatobiliary inflammation.^20^ While no genetic data are available for our AIP–IBD cohort, data from PSC cohorts suggest that this association can be due to similarities in genetic background between PSC and UC.^21^ In the case of AIP and IBD, shared lymphocyte homing mechanisms have been suggested.^22^ Nevertheless, these hypothetical pathophysiological associations should be further explored in well-planned international collaboration studies.

Importantly, we have shown that 20% of the patients with UC and AIP underwent colectomy during a mean follow-up time of 7 years. This rate is higher than that reported in cohorts of UC without AIP,^23,24^ demonstrating a 10-year cumulative colectomy rate of 6–10%. More recent data from the biologics era showed that the cumulative probability of surgery in UC after 5 years from diagnosis was 4.1%.^25^ Our data are in agreement with the GETAID-AIP cohort, identifying AIP–IBD as an independent risk factor for colectomy in both UC and CD patients relative to IBD alone.^9^ Higher rates of colectomies among patients with UC and concomitant AIP were also reported by Hart *et al*.^26^ Taken together, AIP in a patient with IBD should be considered a negative prognostic factor that might be associated with an increased risk for colectomy, particularly in patients with UC.

This series also allows for a detailed characterization of AIP in patients with IBD. Similarly to previous data, we report the predominance of type 2 AIP and abdominal pain being the most frequent initial clinical symptom.^5,9,19^ Interestingly, in 10% of cases, the disorder can be asymptomatic. We also demonstrated that AIP diagnosis preceded that of IBD only in a minority of the cases, confirming the previous report.^9^

As opposed to the complicated IBD course in patients with AIP–IBD, most patients experienced an uncomplicated AIP course. The majority of patients with AIP respond to a single course of steroids. The long-term outcomes of our cohort were also relatively favourable and only one-third experienced one or more episodes of AIP relapse during follow-up. In these patients, subsequent courses of steroids, thiopurines, methotrexate, and anti-tumour necrosis factor alpha agents were used.

The cumulative risk of pancreatic endocrine and/or exocrine insufficiency after a mean AIP follow-up of 5 years was not negligible and reached 26%. To the best of our knowledge, this is the first study defining prognostic factors for the AIP course among patients with IBD. We found in a multivariate analysis that younger age at AIP diagnosis, a family history of IBD, and CD diagnosis were independently associated with a lower risk of developing AIP complications. These data suggest that colonic [UC-type] inflammation is associated with an increased probability of developing pancreaticobiliary disease. Notably, our study found that having a family history of IBD had a protective effect against developing complicated AIP. This finding partially agrees with data from the GETAID-AIP cohort, which showed that patients with concomitant AIP had fewer first-degree family members with IBD compared to those without AIP in univariate models for both UC and the whole IBD group.^9^ Taken together, the results from both studies suggest that having a family history of IBD may reduce the risk of developing AIP or experiencing a more severe course of the disease. Further research, including studies examining the genetic basis of these associations, is needed to better understand the underlying mechanisms.

A few limitations should be noted. First, a retrospective series is subjected to selection and geographical biases, as well as a reporting bias. To address this limitation, we included data from over 30 centres from three continents, thus maximizing the generalizability of our results. Second, we could not compare our cohort directly to non-AIP IBD cases. However, we did compare our results to previously published case-control studies. Finally, external validation of the predictive model was not possible.

In conclusion, this study, the largest international cohort of patients with concomitant IBD and AIP, confirmed the predominance of type 2 AIP and the favourable response to steroid treatment in most cases. It was observed that AIP may be associated with a colonic predominant IBD phenotype, which has a relatively high colectomy rate. Furthermore, a subgroup of patients with a complicated AIP course was identified, mainly consisting of individuals with UC or IBDU, an older age at AIP diagnosis, and no family history of IBD. Further research is needed to explore possible aetiological associations between IBD and inflammatory autoimmune pancreatic involvement.

## Supplementary Material

jjad097_suppl_Supplementary_Table_S1

jjad097_suppl_Supplementary_Table_S2

jjad097_suppl_Supplementary_Table_S3

jjad097_suppl_Supplementary_Table_S4

## Data Availability

The anonymized data underlying this article were provided by the contributing authors. All data except the results cannot be shared publicly. The data underlying this article will be shared on reasonable request to the corresponding authors.
