# Correction to: Autoimmune Pancreatitis in Patients with Inflammatory Bowel Disease: A Real-World Multicentre Collaborative ECCO CONFER Study

**DOI:** 10.1093/ecco-jcc/jjaf077

**Published:** 2025-05-19

This is a correction to: Piotr Eder, Bram Verstockt, Emma Culver, Gabriele Dragoni, Lea Isabell Kredel, Joanna Wypych, Ana Garcia Garcia de Paredes, Magdalena Kaniewska, Haim Leibovitzh, Triana Lobaton, Marie Truyens, Grzegorz Oracz, Davide Giuseppe Ribaldone, Teresa Starzyńska, Abdenor Badaoui, Jean-Francois Rahier, Cristina Bezzio, Peter Bossuyt, Katherine Falloon, Daniela Pugliese, Catherine Frakes Vozzo, Tine Jess, Lone Larsen, Søren Schou Olesen, Partha Pal, María Chaparro, Dikla Dror, Pierre Ellul, Iga Gromny, Maria Janiak, Katarzyna Maciejewska, Noam Peleg, Ariella Bar-Gil Shitrit, Łukasz Szwed, Renata Talar-Wojnarowska, Yifat Snir, Roni Weisshof, Eran Zittan, Izabela Miechowicz, Idan Goren, Autoimmune Pancreatitis in Patients with Inflammatory Bowel Disease: A Real-World Multicentre Collaborative ECCO CONFER Study, *Journal of Crohn’s and Colitis*, Volume 17, Issue 11, November 2023, Pages 1791–1799, https://doi.org/10.1093/ecco-jcc/jjad097

In the version originally published, the second author was incorrectly listed as Bram Verstock. The article has been corrected to list Bram Verstockt.

