# Supplementary material for: Autoimmune Pancreatitis in Patients with Inflammatory Bowel Disease: A Real-World Multicentre Collaborative ECCO CONFER Study
Source: J Crohns Colitis. 2023 Jun 7;17(11):1791–9. doi: 10.1093/ecco-jcc/jjad097 (PMC10673810; doi:10.1093/ecco-jcc/jjad097)
Supplement: jjad097_suppl_Supplementary_Table_S1 [file jjad097_suppl_supplementary_table_s1.doc]

Supplementary Table 1.Countries participating in the study and the number of patients included in the analysis per country.

| Country | Number of patients included |
| --- | --- |
| Belgium  Poland  Italy  United Kingdom  Denmark  Israel  USA  Germany  Spain  Canada  India  Malta | 24  20  13  8  6  6  5  4  4  3  2  1 |
