# Supplementary material for: Autoimmune Pancreatitis in Patients with Inflammatory Bowel Disease: A Real-World Multicentre Collaborative ECCO CONFER Study
Source: J Crohns Colitis. 2023 Jun 7;17(11):1791–9. doi: 10.1093/ecco-jcc/jjad097 (PMC10673810; doi:10.1093/ecco-jcc/jjad097)
Supplement: jjad097_suppl_Supplementary_Table_S2 [file jjad097_suppl_supplementary_table_s2.doc]

Supplementary Table 2. Diagnostic modalities used to confirm histopathological diagnosis of autoimmune pancreatitis in patients with inflammatory bowel disease.

| **Diagnostic modality** | **Frequency of use** |
| --- | --- |
| EUS-FNB | 23/40 (58%) |
| Surgical (pancreaticoduodenectomy due to suspicion of pancreatic cancer) | 7/40 (17%) |
| EUS-guided Trucut biopsy | 6/40 (15%) |
| Biopsy of the papilla of Vater | 2/40 (5%) |
| Cholangioscopy | 1/40 (2.5%) |
| Percutaneous biopsy | 1/40 (2.5%) |
| Abbreviations: EUS-FNB – endoscopic ultrasound-guided fine needle biopsy. | |
