# Supplementary material for: Autoimmune Pancreatitis in Patients with Inflammatory Bowel Disease: A Real-World Multicentre Collaborative ECCO CONFER Study
Source: J Crohns Colitis. 2023 Jun 7;17(11):1791–9. doi: 10.1093/ecco-jcc/jjad097 (PMC10673810; doi:10.1093/ecco-jcc/jjad097)
Supplement: jjad097_suppl_Supplementary_Table_S3 [file jjad097_suppl_supplementary_table_s3.docx]

Supplementary Table 3. The clinical efficacy of the therapy in the subgroup of 18 patients, who received advanced treatment for autoimmune pancreatitis.

| **Type of medication (number of patients treated)** | **Clinical status of autoimmune pancreatitis at the end of follow-up (number of patients)** | **Comment** |
| --- | --- | --- |
| Thiopurines (n=16) | Remission (n=15)  Active disease (n=1) | In 2 cases thiopurines were clinically effective, but had to be stopped due to side effects. Among these 2 patients, anti-tumour factor agent was started with good clinical effect. |
| Methotrexate (n=1) | Remission (n=1) |  |
| Anti-tumour necrosis factor antibody (n=3) | Remission (n=3) | In 2 patients anti-tumour necrosis factor agent was started due to intolerance of thiopurines (see above).  In 1 patient, anti-tumour necrosis factor agent was started as an initial therapy. |
