# Supplementary material for: Autoimmune Pancreatitis in Patients with Inflammatory Bowel Disease: A Real-World Multicentre Collaborative ECCO CONFER Study
Source: J Crohns Colitis. 2023 Jun 7;17(11):1791–9. doi: 10.1093/ecco-jcc/jjad097 (PMC10673810; doi:10.1093/ecco-jcc/jjad097)
Supplement: jjad097_suppl_Supplementary_Table_S4 [file jjad097_suppl_supplementary_table_s4.doc]

Supplementary Table 4. Clinical, biochemical and radiological worrisome features in patients with suspected autoimmune pancreatitis [3,8,16-18]

| **Clinical and biochemical worrisome feature** | **Radiological worrisome features** |
| --- | --- |
| Weight loss | Focal pancreatic mass |
| Debilitating pain | Dilatation of main pancreatic duct (especially secondary to a regular, single and short stricture) |
| New onset or worsening of pre-existing diabetes | Distal pancreatic parenchymal atrophy |
| Jaundice (especially stable or progressing in time) | Lack of delayed contrast enhancement of an enlarged pancreas with no capsule-like rim on dynamic computed tomography |
| Elevated CA 19-9 | Higher values of the apparent diffusion coefficient values on diffusion-weighted magnetic resonance |
| No elevation in IgG4 serum levels | Lack of radiological manifestation suggesting systemic IgG4-related disease |
| Lack of clinical manifestations suggesting systemic IgG4-related disease | Lack of radiological response to a short trial of steroids* |
| Lack of clinical response to a short trial of steroids* |  |

*the duration of a steroid trial varies between 2 – 6 weeks according to different recommendations [8,17,18]
